# Effects of *Moringa oleifera* aqueous seed extracts on reproductive traits of heat-stressed New Zealand white female rabbits

**DOI:** 10.3389/fvets.2022.883976

**Published:** 2022-09-12

**Authors:** Valence B. Mutwedu, Albert W. Nyongesa, Jafred M. Kitaa, Rodrigue B. B. Ayagirwe, Chasinga Baharanyi, James M. Mbaria

**Affiliations:** ^1^Department of Animal Production, Faculty of Agriculture and Environmental Studies, Université Evangélique en Afrique (UEA), Bukavu, Democratic Republic of Congo; ^2^Department of Public Health, Pharmacology and Toxicology, Faculty of Veterinary Medicine, University of Nairobi, Nairobi, Kenya; ^3^Department of Veterinary Anatomy and Physiology, Faculty of Veterinary Medicine, University of Nairobi, Nairobi, Kenya; ^4^Clinical Studies Department, Faculty of Veterinary Medicine, University of Nairobi, Nairobi, Kenya; ^5^Department of Pathology, Faculty of Medicine, Université Evangélique en Afrique (UEA), Bukavu, Democratic Republic of Congo

**Keywords:** *Moringa oleifera*, oxidative stress, reproduction, female rabbits, heat stress

## Abstract

Heat stress is reported to have deleterious effects on rabbit physiology by impairing reproductive performances arising from free radical production due to oxidative stress. Plant extracts have been listed among efficient and healthy strategies for alleviating the effects of free radicals in the body of an animal. Numerous studies have documented the medicinal value of *Moringa oleifera* on various body functional systems although most of these data have not been scientifically validated. The growing concern of heat stress owing to the effects of global warming has affected animal productivity and even reproductive health, yet mitigation measures are still scanty. To this end, we investigated the efficacy of *Moringa oleifera* aqueous seed extract on selected in the alleviation of morphofunctional impairments on functional systems of the body. Here, we quantified the effects of *Moringa oleifera* seed extracts on reproductive performances, hormonal profile, and ovarian histology in the management of heat stress in female rabbits. We were particularly interested in testing the hypothesis that *Moringa oleifera* seed extracts do not have medicinal value in the mitigation of oxidative stress accompanying heat-stressed animals and, therefore, affecting growth performance and reproductive value. Twenty-eight female rabbits aged 6 months and weighing between 2015.6 and 2322.7 g were randomly assigned to four treatment groups of temperature, relative humidity, temperature humidity index, and M. oleifera seed extracts as follows: T0: ambient temperature (18–24°C), 59 ± 0.48%, 23.6 ± 1.52; T1, T2, and T3: 35–36°C, 64 ± 0.6%, 32.5 ± 0.7, followed by 100, 200, and 0 mg/kg b.w of M. oleifera seed extracts. Artificial heat was introduced in each rabbit cage from 08:00 h to 16:00 h. Reproductive performances related to female rabbits and relevant biodata of their young ones were recorded. All animals were sacrificed at the end of 80 days of experiments, and blood was collected for hormonal assays and ovary tissues for histology. Data on hormones and reproductive parameters of adult females as well as parameters related to kid performance and milk yield were subjected to one-way ANOVA, and significant differences among treatment subjects were analyzed using Tukey's *post-hoc* test at 5% significance level. The results revealed a significant decrease (*P* < 0.05) in food consumption, body weight, and body weight gain in pregnant and lactating female rabbits exposed to heat stress. A decrease in fur removal by mothers, litter size from birth to weaning, litter weight, kid body weight, and body weight gain in adult rabbits submitted to heat stress was observed. The findings were also reflected on weekly milk yield and daily milk efficiency as well as serum hormone levels. Following administration of M. oleifera seed extracts at 200 mg/kg b.w., there was significant increase (*P* < 0.05) in these parameters. On the contrary, an increase (*P* < 0.05) in the number of services per conception, milk intake, and serum progesterone level was initially observed in the same subjects, but upon administration of M. oleifera seed extracts, there was a significant decrease (*P* < 0.05) on these measures. Ovarian histology of animals at T0 and T2 treatments showed structural features comparable to those of controls. Overall, our results show that administration of *M. oleifera* seed extracts at 200 mg/kg b.w possesses therapeutic value to the effects of oxidative stress associated with heat stress. Further pharmacological evaluation on seed extracts of *M. oleifera* may yield the much-needed medicine in the treatment and management of poor animal productivity and reproductive health arising from severe weather associated with global warming.

## Introduction

Heat stress has been defined as a heat load sustained under the combined effect of environmental factors (air temperature, humidity, airflow, and heat radiation) and metabolic heat production (1). It occurs when the environmental temperature exceeds the thermoneutrality zone of the animal (2). Heat stress alters animals' physiology, with the reproduction as the first function to be impaired (3). In fact, heat stress has been reported to impair embryo development by increasing their mortality in cattle (4). It is associated with the low fertility rate in dairy cows by poor expression of estrus due to a reduced estradiol secretion (5).

Rabbits are reported as one of the animals most affected by thermal stress (6, 7) since they do not have enough sweat glands to allow dissipation of excess body heat (8). Rabbit thermoneutrality zone ranges between 16 and 21°C, and above this range, physiological, productive, and reproductive performances begin to decrease (2). Marai et al. (6) reported a decrease in rabbit weight, litter size, litter weight at weaning, conception rate, and an increase in pre-weaning mortality in adult females exposed to severe heat stress during summer. A decrease in progesterone and estradiol levels has been reported in female rabbits exposed to 41°C (9). Meanwhile, rabbits exposed to the temperature between 25 and 36°C compared to those maintained between 14 and 20°C decreased their litter size (9.7 vs. 11.4), litter weight (503.0 vs. 630.5 g), and kit weight at birth (56.6 vs 61.4 g) and increased the stillbirth rate (25.4 vs. 9.9%) during pregnancy and lactation (10).

However, considering the economic benefits and social considerations of the rabbit under the tropics (11, 12), it is therefore important to look for alternative solutions to minimize the effect of heat stress on its productive and reproductive performances to enhance farmers' income (12, 13).

Phytogenic feed supplements are plant-derived products used in animal feeds to improve the agricultural livestock performance (14). They are preferred because of their high antioxidant capacity, cheap availability, and low toxicity and are ecological friendly for the treatment of various ailments (14, 15).

*Moringa oleifera*, a perennial tree growing up to between 5 and 12 m high and belonging to the Moringaceae family, is considered as one of the best antioxidant plants worldwide (16). All parts of this plant including leaves, stembarks, pods, and seeds are reported to be rich in various bioactive compounds such as alkaloids, saponins, phenols, flavonoids, glycosides, terpenoids, and tannins (17–19). Due to the presence of these compounds, *Moringa oleifera* has been therefore reported to alleviate damages caused by oxidative stress (20, 21). Odeyinka et al. (22) reported an improvement in litter size, litter weight, gestation length, and milk yield following supplementation of leaves of *Moringa oleifera*. In a study that included 10% of *Moringa oleifera* in female rabbits' meal, the litter weight at birth, the litter weight, the weaning weight, and the survival rate were improved (23). An improvement in reproductive hormones such as LH, FSH, estrogen, progesterone and prolactin was reported in female rabbits fed with 5, 10, and 15 g/kg of *Moringa oleifera* leaf powder incorporated into rabbit grower's pellet (24). All these findings were performed using *Moringa oleifera* leaves that have been reported to possess better antioxidant activity (15) and therefore overused compared to other parts of the plant. It is, therefore, important to explore the effectiveness of other parts of this plant for medicinal value in overcoming health challenges associated with oxidative stress in animals. However, the pharmacological efficacy of seeds of *Moringa oleifera* on reproductive parameters of female rabbits exposed to heat stress is scant in literature.

It is hypothesized that *Moringa oleifera* aqueous seed extracts have no efficacy on the heat stress damage leading to impaired reproductive performances, altered ovarian histology, and reproductive hormonal profile of female rabbits. This study was designed to evaluate the efficacy of seeds of *Moringa oleifera* aqueous extract on reproductive performances, hormonal profile, and ovary histology in the management of heat stress in female rabbits.

## Materials and methods

### Experimental location

The study was conducted from September to December 2019 at the Department of Veterinary Anatomy and Physiology, University of Nairobi, Kenya. The average temperature was 24°C, and the relative humidity was generally greater than 58%.

### Plant extract

The seeds of *Moringa oleifera* (Lam) aged 2 to 3 years were collected from Masii village of Machakos County in Kenya. The plant was taxonomically identified at the Department of Biology and Biotechnology, University of Nairobi, Kenya, and a voucher specimen (MK032) was deposited in the herbarium at the School of Biological Sciences, University of Nairobi, Chiromo Campus. The seeds were air-dried at 21 to 25°C for 14 days. After spreading, the plant material was ground using an electric mill and the fine powder was collected and stored in airtight glassware until aqueous extraction.

The aqueous extract was obtained using the method described by Okumu et al. (25). Briefly, a total of 1,000 g of dried *Moringa oleifera* seed powder were aliquoted into a vial wrapped in aluminum foil. Ten l of distilled water was gradually added to the powder with gentle stirring using a magnetic wand until a slurry of uniform consistency was formed. The formed slurry was further stirred using a magnetic stirrer operating at 200 RPM for 48 h. The resulting slurry was centrifuged at 3,000 RPM for 5 min, and the filter media were collected for further processing.

The bioactive compounds were detected using phytochemical tests according to standard methods developed by Harborne (26). For quantitative analysis, the concentration of different compounds mainly alkaloids, saponins, flavonoids, total phenols, glucosides, terpenoids, and tannins was evaluated following the methods developed, respectively, by Harborne (27), Ejikeme et al. (28), Edeoga et al. (29), Amadi et al. (30), Ferguson (31), and Van-Burden and Robinson (32). Table 1 shows the bioactive compounds detected in *Moringa oleifera* seed aqueous extract.

**Table 1:** Percentage of crude phytochemicals in aqueous extract of *Moringa oleifera* seed collected from Machakos County.

**Compounds (%)**	**Aqueous extract**
Alkaloids	5.56
Saponins	-
Phenols	3.88
Flavonoids	0.53
Glycosides	4.18
Terpenoids	0.28
Tannins	**-**

### Experimental animals and their feeding

A total of 28 female rabbits, aged 6 months and weighing 2,286.1 ± 63.5 g (Mean ± SE), purchased from a local recognized and licensed breeder, were used. Throughout the experimental period, feed and water were provided *ad libitum* to animals. All animals used for the experiment were provided with the basal commercial pelleted ration containing 18.18% crude protein, 13.43% crude fiber, 2,656 MJ/kg diet digestible energy, and 2.29% ether extract that met all nutritional requirements of rabbit does according to the National Research Council (33). The rabbit cages were routinely cleaned every morning before clinical observations to the animals.

### Experimental design

During the acclimatization period, the rabbits were housed in wire cages (0.8 × 0.6 × 0.6 m) at room temperature of 21 ± 3°C with animal house relative humidity of 66 ± 3% and kept under the same hygienic and managerial conditions. Before starting the experiment, the animals were weighed, randomly assigned to four groups of seven female rabbits each with comparable body weight, and distributed to T0 and T3 (normal and positive control, respectively) and T1 and T2 (low and high doses of *Moringa oleifera*, respectively). Thereafter, during 80 consecutive days, animals were submitted to different room temperatures, relative humidity, temperature humidity index (THI), and of *Moringa oleifera* aqueous seed extract (MOASE) as follows: T0: ambient temperature (18–24°C), 59 ± 0.48%, 23.6 ± 1.52; T1: 35–36°C, 64 ± 0.6%, 32.5 ± 0.7, 100 mg/kg b.w of MOASE; T2: 35–36°C, 64 ± 0.6%, 32.5 ±0.7, 200 mg/kg b.w of MOASE; T3: 35–36°C, 64 ± 0.6%, 32.5 ± 0.7. The heat was induced in each rabbit cage, using electrical heaters (brand: ARMCO from India) from 08:00 h to 16:00 h followed by exposure to the normal air temperature as in the control group from 16.00 h to 08:00 h. During the experimental period, the relative humidity and ambient temperature were recorded twice daily using an automatic thermo-hygrometer (Brand: RC dalys, Size: 48^*^28.6^*^15.2 cm, temperature precision: ±1°C, hygrometry precision: ±5). The selected range of temperature, relative humidity, and THI was chosen according to the results observed in our previous study (34) and was classified as very severe heat stress (35). *Moringa oleifera* aqueous seed extracts (MOASE) were administrated per os once a day for 80 days using an endogastric cannula, while normal and negative control animals (T0 and T3, respectively) orally received 10 ml of distilled water daily. 2 weeks after submitting animals to heat stress and gavaging *Moringa oleifera* aqueous seed extract, each female rabbit was transferred in a single proven fertile males' cage for mating. 10 days after mating, pregnancy was evaluated by abdominal palpation by hands feeling around until finding of little lumps that can pass through fingers as gently probe and search (35). The females failing to conceive were immediately returned to the same buck for another service.

In pregnant and lactating female rabbits, feed intake (F.I.) and weight gain (W.G.) were measured daily and daily body gain was calculated for each female rabbit according to the method described by Sabah and Dalal (9).

### Reproductive performances

The gestation length was obtained by calculating the duration (in days) between the date of the coupling and the date of farrowing (36, 37). The conception rate was estimated as number of services per conception, and the type of mating observed was classified as voluntary mating (i.e., mating receptive does) and hand mating (i.e., mating unreceptive does) (35). The litter size was obtained by counting the number of young rabbits of each female at farrowing and weekly until weaning (36–38). The stillbirth rate was evaluated as the number of stillborn young rabbits on the litter size at birth (38), and the survival rate was calculated as the litter size at weaning on the litter size at birth (37, 38). The young rabbit weight evolution was obtained by weighing young rabbits at birth and every week until 5 weeks post-partum (weaning) (36–38).

For each female, milk yield was evaluated as the difference in weight of the kids before and after suckling. Milk intake per kit was calculated as the milk yield on the number of kits in the litter for each female rabbit, and the efficiency of converting milk into body weight gain was calculated as the kit weight gain on the milk intake per kit (35).

The litter weight was evaluated by weighing the whole kids of the litter, the kit body weight was estimated by weighing separately each kid, and the kit body weight gain was evaluated as the difference between the actual kit weight and the kit weight of the previous week (35). These parameters were estimated at weekly intervals between birth and 35 days of age (weaning).

### Sacrifice of rabbits, blood collection, and processing

At the end of the experimental period (80 days), all animals were fasted for 24 h and humanely sacrificed by euthanizing using ether vapor and then dissected. Ten ml (10 ml) of the blood was collected directly by cardiac puncture before euthanasia using ether vapor by inhalation. The obtained blood was then centrifuged, and the resultant serum was kept at −20°C for hormones assessment. After sacrifice, the ovaries were collected by dissection, freed of adipose tissue, washed using saline solution, and blot-dried for histology.

### Hormone evaluation

The principal hormones analyzed were as follows: estradiol, follicle-stimulating hormone (FSH), luteinizing hormone (LH), progesterone, prolactin (PL), and cortisol. Cortisol was estimated by the radioimmunoassay (RIA) technique using coated tubes kits (Diagnostic Systems Laboratories, Inc. Webster, Texas 77598-4217, USA) and the tracer labeled with iodine-125 (I^125^). Quantitative determination of serum estradiol, FSH, LH, progesterone, and PL was performed using the solid-phase enzyme-linked immunosorbent assay (ELISA) as described by the commercial kit Omega Diagnostic (23961 Craftsman Road, Suite D/E/F, Calabasas, CA 91302). Briefly, 100 uL of samples was added to the appropriate well in the antibody pre-coated Microtiter Plate. Then, 100 uL of PBS (pH 7.0–7.2) was added in the blank control well; 10 uL of Balance Solution was dispensed into 100 uL specimens and then mixed well; 50 uL of the conjugate was added to each well-except the blank control well and then mixed before incubation for 1 h at 37°C. After incubation, the plate was washed using manual washing. The incubation mixture was removed by aspirating contents of the plate into a sink; then, the plate was washed five times with a wash solution. After washing, invert the plate and blot dry by hitting the plate onto absorbent paper until no moisture appears. About 50 uL of Substrate A and 50 uL of Substrate B were added into each well-including blank control well, subsequently, and then incubated for 10–15 min at 20–25°C. After incubation, 50 uL of Stop Solution was added to each well-including blank control well. Therefore, the optical density (O.D.) was determined at 450 nm using a microplate reader immediately. The standard curve was used to determine the concentration of samples as corresponding to the mean absorbance from the standard curve.

All analyses were performed following the manufacturers' recommendations.

### Ovary histology

Immediately after sacrifice, the left ovary of each female rabbit was removed, fixed in 10.00% formalin for 1 week, then washed, dehydrated with ascending grades of alcohol bath, clarified in xylene immersion, embedded in paraffin, cut at 5.00-μm thickness, and stained with hematoxylin and eosin. The sections were observed under a light microscope (Leica DM 750, X10, and X40) equipped with a DCM35 digital camera (350 Kpixels, USB 2.0), which was connected to the central processing unit of a computer. The images were directly observed on the computer equipped with an image capture program. The captured images were magnified 100 times (100X) for histology and cellular integrity.

### Statistical analysis

Data were compiled into a database using Excel package. For the analysis of the different treatments, the effects among experimental groups and control were assessed using one-way ANOVA. The differences in mean values were compared using the Tukey HSD test at P < 0.05. Data analysis was performed using XLSTAT for Windows 10 Software. The results are expressed as the means ± SD, graphs, and charts. The effect rate (ER) was calculated as the ratio between the value of the control group and the value of the group with the most relevant impact.

## Results

### Growth performances in pregnant and lactating female rabbits

**Figure 1:**
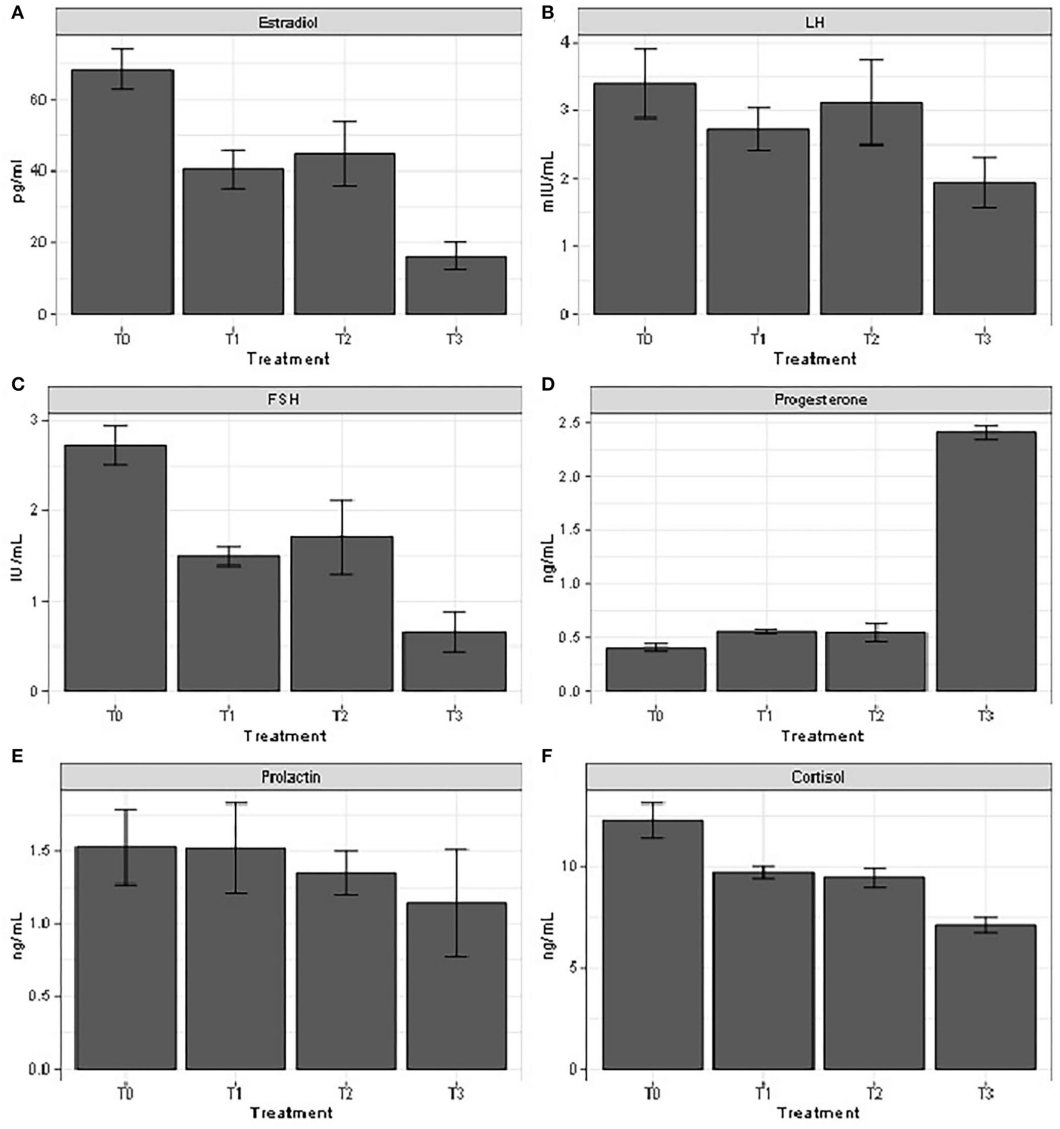
Reproductive hormones profile following administration of different doses of the Moringa oleifera aqueous seed extract in female rabbits exposed to heat stress. T0: control group, T1: 35−36°C + 100 mg MO, T2: 35−36°C + 200 mg MO, T3: 35−36°C. LH: Luteinizing Hormone; FSH: Follicular Stimulating Hormone; n = 7.

The results on feed consumption (Figure 1) showed decrease of 21 and 34%, respectively, in pregnant (A) and lactating (B) female rabbits submitted to heat stress compared to those of the control group. However, in *Moringa oleifera* aqueous seed extract-treated animals, this parameter increased in a dose-dependent manner. Similar results have been reported in body weight of both pregnant (C) and lactating (D) female rabbits by 8 and 26%, respectively, except at the 5th week where body weight of animals submitted to heat stress and treated with 200 mg/kg b.w. of *Moringa oleifera* aqueous seed extract. In these animals, body weight was slightly higher by 4% compared to that of the control group in pregnant females (C) and of animals submitted to heat stress and treated with 100 mg/kg b.w. of *Moringa oleifera* aqueous seed extract which suddenly decreased by 3% in lactating females (D). There was a dose-dependent effect of *Moringa oleifera* aqueous seed extract on body weight gain in pregnant females (E) except at the 2nd week where animals of the control group and those submitted to heat stress alone showed a greater body weight gain. However, in lactating females (F), the animals of the control group showed the highest body weight gain (8%) compared to those submitted to heat stress, except at the 1st week of lactation where animals treated with 200 mg/kg b.w. of *Moringa oleifera* aqueous seed extract had the highest body weight gain. In these animals, body weight gain decreased weekly until weaning while animals solely submitted to heat stress showed loss in body weight from the 2nd week of treatment.

### Reproductive performances in female rabbits

The results of Table 2 showed that voluntary mating was the most observed type of mating in female rabbits even though hand mating increased with the heat stress. Generally, most of animals of the control group required one service to achieve pregnancy (71.4%), while 42.9% of animals submitted to heat stress and co-treated with 100 and 200 mg/kg b.w. *Moringa oleifera* aqueous seed extract, respectively, required two services to achieve pregnancy and most of animals submitted to heat stress alone required two services to become pregnant (71.4%). The self fur removal was intense in most animals of control group (71.4%), lightly intense in animals co-treated with 100 mg/kg b.w. (57.1%) and 200 mg/kg b.w. *Moringa oleifera* aqueous seed extract (85.7%), while most of animals of the group solely submitted to heat stress did not remove their fur (57.1%). A significant decrease in the litter size was observed in animals submitted to heat stress compared to those of control group. However, this parameter increased in a dose-dependent manner following administration of *Moringa oleifera* aqueous seed extract. There was no group significant difference in the gestation length.

**Table 2:** Reproductive performances following administration of different doses of the *Moringa oleifera* aqueous seeds extract in female rabbits exposed to heat stress.

**Parameters**	**T0**	**T1**	**T2**	**T3**
**Type of mating (%)**				
Voluntary mating	85.7	71.4	71.4	71.4
Hand mating	14.3	28.6	28.6	28.6
**Number of services per conception (%)**				
One	71.4	57.1	57.1	28.6
More	28.6	42.9	42.9	71.4
**Self plucked fur (%)**
Dense	71.4	14.3	14.3	0
Light	28.6	57.1	85.7	42.9
Without plucking	0	28.6	0	57.1
Gestation length	30.8 ± 0.3	31.1 ± 0.4	31.1 ± 0.2	31.1 ± 06
Litter size	8.3 ± 1.2^a^	6.2 ± 1.1^b^	7.6 ± 1.4^ab^	5.6 ± 1.3^b^

The superscripts a, ab, b: means the mean values are significantly different at P < 0.05; T0: control group, T1: 35–36°C+100 mg MO, T2: 35–36°C+200 mg MO, T3: 35–36°C; n = 7.

### Litter size at different ages of litter from birth to weaning

The number of kids was significantly higher in female rabbits from the control group from birth to weaning (35 days) compared to those submitted to heat stress. However, in animals submitted to heat stress and co-treated with *Moringa oleifera* aqueous seed extract, this parameter increased in a dose-dependent manner without reaching the values of the control group (Table 3).

**Table 3:** Litter size at different ages of litter from birth to weaning following administration of different doses of the *Moringa oleifera* aqueous seeds extract in female rabbits exposed to heat stress.

**Parameters**	**T0**	**T1**	**T2**	**T3**	***p*-value**
Birth	8.3 ± 1.2^a^	6.2 ± 1.1^b^	7.6 ± 1.4^ab^	5.6 ± 1.3^b^	0.029*
7 days	7.2 ± 0.3^a^	4.4 ± 0.3^b^	6.5 ± 1.6^a^	4.1 ± 0.2^b^	0.044*
14 days	6.9 ± 0.3^a^	4.4 ± 0.3^c^	5.8 ± 0.5^b^	3.5 ± 0.3^d^	<0.001***
21 days	6.9 ± 0.3^a^	3.5 ± 0.3^c^	4.5 ± 0.4^b^	2.7 ± 0.2^d^	<0.001***
28 days	6.9 ± 0.3^a^	2.9 ± 0.2^c^	4.0 ± 0.3^b^	1.6 ± 0.2^d^	<0.001***
35 days	6.9 ± 0.3^a^	2.9 ± 0.2^c^	4.0 ± 0.3^b^	1.6 ± 0.2^d^	<0.001***

The superscripts a, b, c, d: means the mean values are significantly different at P < 0.05; T0: control group, T1: 35–36°C+100 mg MO, T2: 35–36°C+200 mg MO, T3: 35–36°C. ^*^, ^***^: significant at P < 0.05 and 0.001, respectively; n = 7.

### Litter weight, kit body weight, and kit body weight gain from birth to weaning

The results of Table 4 showed a significant increase in litter weight of the control group from birth to weaning (35 days) compared to groups submitted to heat stress. However, when co-treated with *Moringa oleifera* aqueous seed extracts, this parameter increased in a dose-dependent manner but without achieving the values of the control group. The kit body weight at weaning (35 days) and the daily kit weight gain indicated a significant increase in the control group compared to groups submitted to heat stress. Nevertheless, in heat-stressed animals, these parameters were increased in female rabbits co-treated with 100 and 200 mg/kg b.w. *Moringa oleifera* aqueous seed extract, respectively, compared to those submitted to heat stress alone.

**Table 4:** Evolution of the litter weight, kit body weight and kit body weight gain from birth to weaning following administration of doses of the *Moringa oleifera* aqueous seed extract in female rabbits exposed to heat stress.

**Parameters**	**T0**	**T1**	**T2**	**T3**	***p*-value**
**Litter weight (kg)**
Birth	332.6 ± 17.3^a^	241.8 ± 18.2^b^	253.5 ± 14.7^b^	218.4 ± 16.3^b^	0.038*
7 days	553.6 ± 26.8^a^	277.2 ± 31.4^c^	449.5 ± 14.7^b^	230.8 ± 12.5^d^	<0.001***
14 days	1092.1 ± 23.1^a^	804.3 ± 14.6^c^	891.7 ± 28.8^b^	351.2 ± 22.3^d^	<0.001***
21 days	1485.7 ± 31.5^a^	616.4 ± 17.7^c^	850.5 ± 23.5^b^	434.7 ± 13.4^d^	<0.001***
28 days	2435.7 ± 31.5^a^	925.1 ± 24.1^c^	1321.9 ± 22.7^b^	476.8 ± 22.7^d^	<0.001***
35 days	3081.8 ± 57.4^a^	1220.9 ± 34.5^c^	1752.1 ± 34.3^b^	592.4 ± 23.7^d^	<0.001***
**Kit body weight (g)**
At birth	41.6 ± 1.3	39.6 ± 1.7	39.2 ± 1.1	39.3 ± 1.5	0.066
35 days	448.7 ± 12.5^a^	428.1 ± 10.4^b^	431.1 ± 6.9^b^	373.2 ± 11.4^c^	0.027*
**Kit weight gain (g/day)**	14.4 ± 0.4^a^	12.8 ± 0.4^b^	13.2 ± 0.7^b^	9.8 ± 0.6^c^	0.034*

The superscripts a, b, c, d: means the mean values are significantly different at P < 0.05; T0: control group, T1: 35–36°C+100 mg MO, T2: 35–36°C+200 mg MO, T3: 35–36°C. ^*^, ^***^: significant at P < 0.05 and 0.001, respectively; n = 7.

### Milk yield, milk intake, and milk efficiency

The results indicate a significant increase in milk yield in female of the control group from birth to weaning (35 days) compared to groups submitted to heat stress. However, in animals submitted to heat stress and co-treated with *Moringa oleifera* aqueous seed extract, this parameter increased in a dose-dependent manner without reaching the values of the control group. The daily kit milk intake of the 1st and 2nd weeks was high in animals of the control group and those receiving 100 and 200 mg/kg b.w. *Moringa oleifera* aqueous seed extract, respectively, compared to those only submitted to heat stress; the opposite trend was observed at the fourth and 5th weeks, while there was not any significant difference at the 3rd week. Except the 3rd week where there was no significant difference, the milk efficiency significantly increased in females of the control group and those co-treated with 100 and 200 mg/kg b.w. *Moringa oleifera* aqueous seed extract, respectively, during the first, second, fourth, and fifth weeks compared to the group submitted to the heat stress alone (Table 5).

**Table 5:** Evolution of the milk yield, milk intake and milk efficiency from birth to weaning following administration of doses of the *Moringa oleifera* aqueous seed extract in female rabbits exposed to heat stress.

**Parameters**	**T0**	**T1**	**T2**	**T3**	***p*-value**
**Milk yield (g per litter per day) per day**
7 days	75.8 ± 2.8^a^	54.6 ± 3.6^c^	68.3 ± 2.5^b^	48.1 ± 3.1^d^	<0.001***
14 days	99.3 ± 8.4^a^	76.4 ± 7.7^bc^	80.6 ± 6.5^b^	65.3 ± 7.1^c^	0.019*
21 days	94.1 ± 9.5^a^	59.8 ± 7.4^c^	71.7 ± 7.9^b^	44.3 ± 6.5^d^	<0.001***
28 days	90.1 ± 7.3^a^	42.6 ± 5.3^c^	53.1 ± 3.8^b^	33.7 ± 4.9^d^	<0.001***
35 days	79.2 ± 5.5^a^	34.7 ± 4.8^c^	46.1 ± 5.9^b^	31.8 ± 3.4^c^	0.033*
**Milk intake (g per litter per day) per day**
7 days	10.6 ± 0.8^a^	8.9 ± 0.5^b^	10.4 ± 1.0^a^	8.6 ± 1.1^b^	0.040*
14 days	14.1 ± 1.0^b^	16.4 ± 1.1^a^	13.8 ± 0.7^b^	14.8 ± 0.4^b^	0.021*
21 days	13.4 ± 1.1	14.3 ± 1.8	14.9 ± 1.3	14.1 ± 0.6	0.081
28 days	13.1 ± 1.5^b^	14.1 ± 1.5^b^	13.3 ± 3.8^b^	19.4 ± 0.2^a^	0.033*
35 days	11.6 ± 0.8^b^	10.9 ± 4.8^b^	11.4 ± 0.6^b^	18.7 ± 0.7^a^	0.029*
**Milk efficiency**
7 days	1.4 ± 0.3^a^	1.4 ± 0.1^a^	1.3 ± 0.1^a^	1.1 ± 0.1^b^	0.040*
14 days	1.0 ± 0.1^a^	0.7 ± 0.1^b^	0.9 ± 0.1^a^	0.6 ± 0.1^b^	0.021*
21 days	1.1 ± 0.2	0.9 ± 0.2	0.8 ± 0.1	0.7 ± 0.2	0.081
28 days	1.1 ± 0.3^a^	0.9 ± 0.2^a^	1.0 ± 0.2^a^	0.5 ± 0.2^b^	0.033*
35 days	1.2 ± 0.2^a^	1.2 ± 0.2^a^	1.2 ± 0.1^a^	0.5 ± 0.2^b^	0.029*

The superscripts a, b, c, d: means the mean values are significantly different at P < 0.05; T0: control group, T1: 35–36°C+100 mg MO, T2: 35–36°C+200 mg MO, T3: 35–36°C. ^*^, ^***^: significant at P < 0.05 and 0.001, respectively; n = 7.

The superscripts a, b, c: means the mean values are significantly different at P < 0.05; T0: control group, T1: 35–36°C+100 mg MO, T2: 35–36°C+200 mg MO, T3: 35–36°C. ^*^: significant at P < 0.05; LH, Luteinizing Hormone; FSH, Follicular Stimulating Hormone; n = 7.

### Reproductive hormones profile

**Figure 2:**
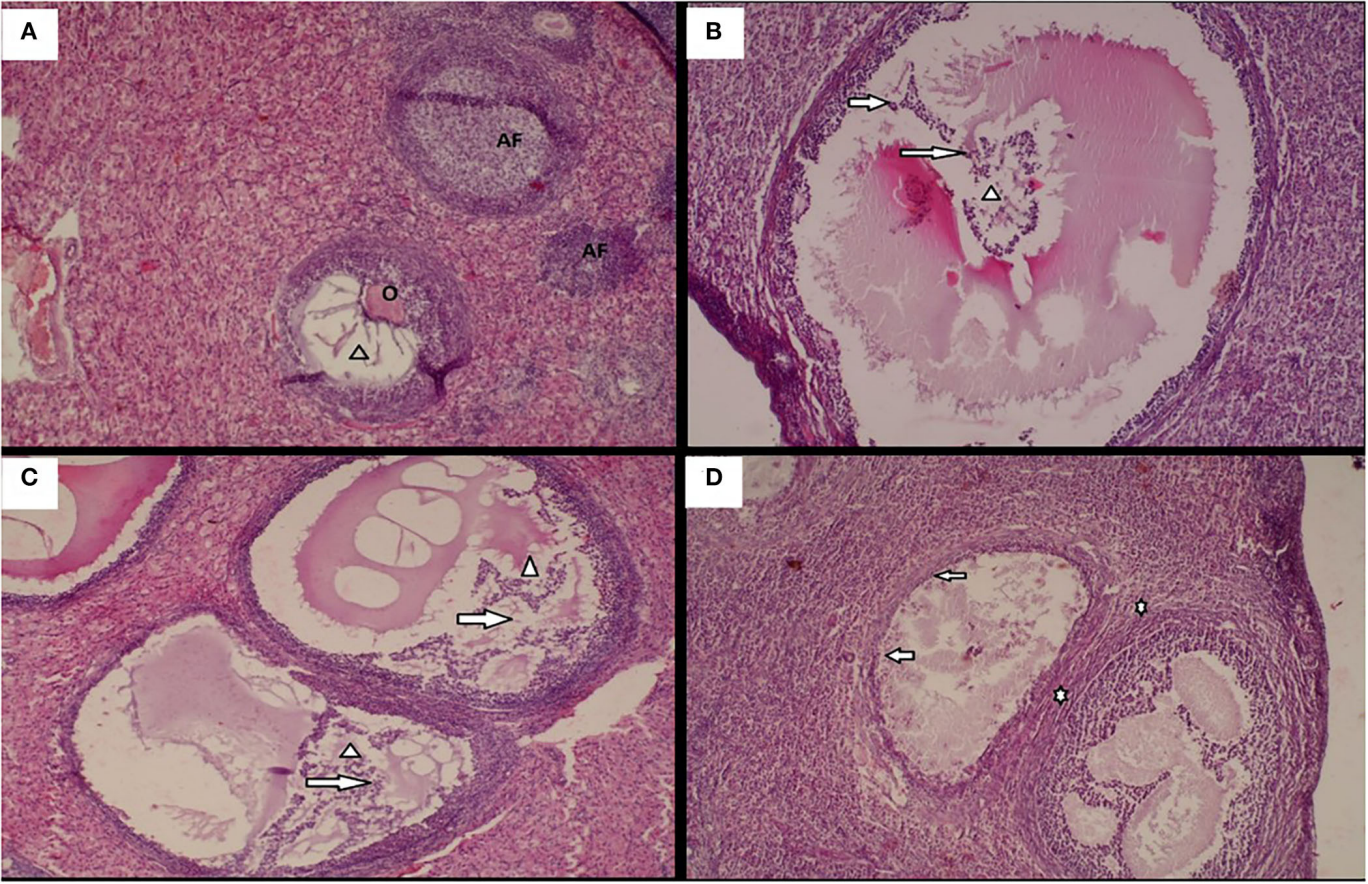
Histological changes in animals following administration of different doses of the Moringa oleifera seeds aqueous extract in female rabbits after exposure to heat stress. **(A)** Ovary with a dominant growing follicle with oocyte **(O)**, forming antral cavity **(Arrow head)**, and surrounded by atretic follicles **(AF)** (H/E X 40). **(B)** Marked degeneration of Graafian follicle with oocyte lysis **(Arrow head)**, severe degeneration of granulosa cells and cumulus oophorous **(Arrow)** (H/E X 100). **(C)** Multifocal areas of follicular degeneration characterized with oocyte lysis **(Arrow head)**, Degeneration of granulosa cells and theca cells **(Arrow)** (H/E X 100). **(D)** Focal areas of proliferation of fibroblast in the interstitial tissues (*), in addition to severe follicular degeneration characterized by oocyte lysis and degeration of follicular cells **(Arrow)** (H/E X 100).

The results of Figure 2 indicated that serum levels of estradiol and FSH significantly decreased in the heat-stressed animals compared to the control group. These parameters increased in heat-stressed animals, co-treated with 100 and 200 mg/kg b.w. *Moringa oleifera* aqueous seed extract, respectively, compared to those submitted to heat stress alone. The opposite trend was observed in serum progesterone and cortisol levels. On the contrary, LH content was decreased (*P* < 0.05) in animals submitted to heat stress alone compared to those of the control group and those receiving 100 and 200 mg/kg b.w. *Moringa oleifera* aqueous seed extract, respectively. There was no significant difference in serum prolactin content.

### Ovarian histology

**Figure 3:**
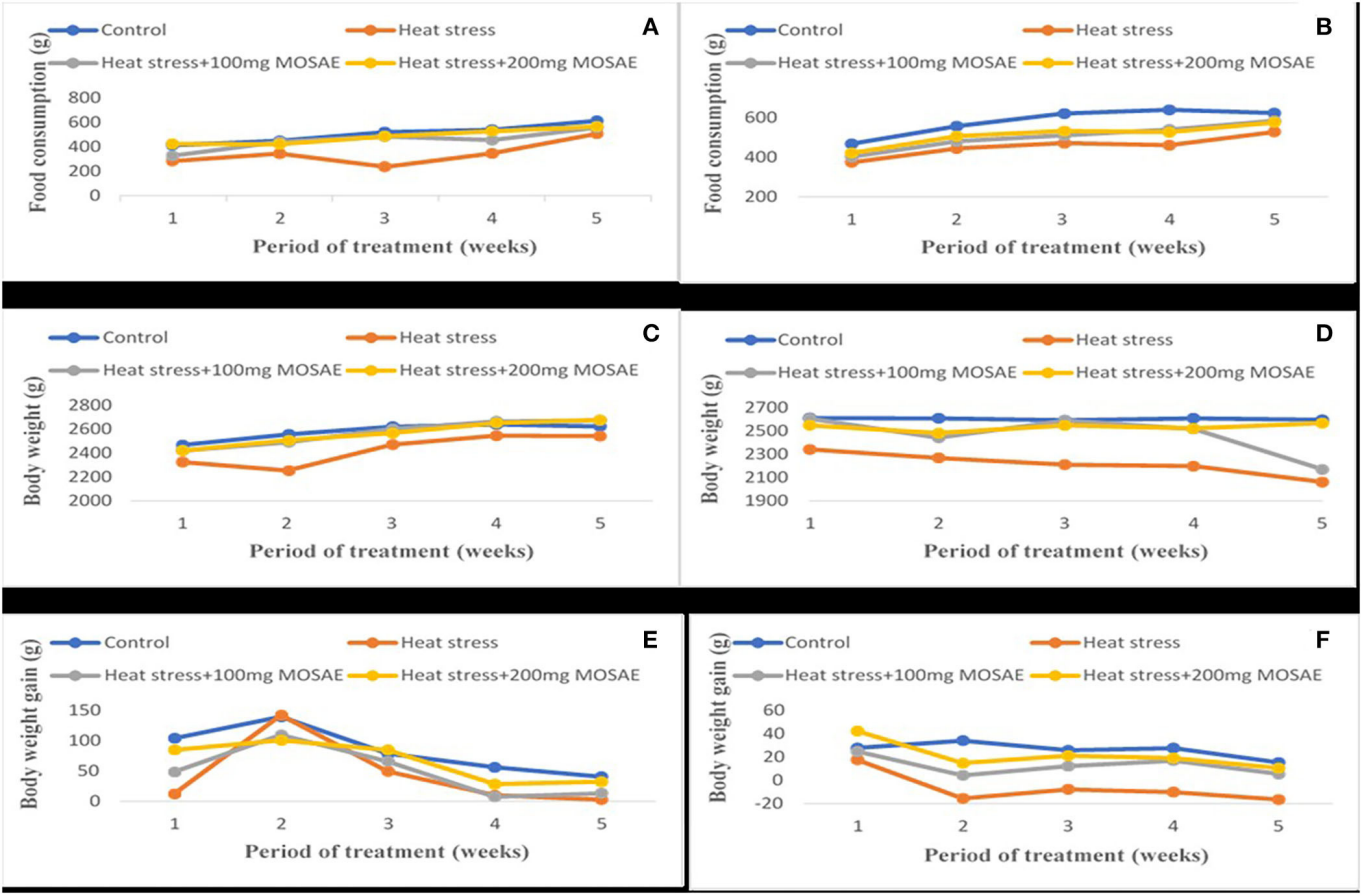
Variation of the food consumption, body weight and body weight gain following different doses of the Moringa oleifera aqueous seed extract in pregnant and lactating female rabbits exposed to heat stress. Control: ambient temperature; Heat stress: 359636°C, MOSAE: Moringa oleifera seeds aqueous extract, **(A–B)** Food consumption in pregnant and lactating female rabbits respectively; **(C–D)** Body weight in pregnant and lactating female rabbits respectively; **(E–F)** Body weight gain in pregnant and lactating female rabbits respectively.

The ovary of animals (Figure 3) from control group **(A)** was characterized by the presence of a dominant growing follicles with oocyte, forming antral cavity, and surrounded by atretic follicles. However, the ovary of animals submitted to heat stress and co-treated with 200 mg/kg b.w. of Moringa Oleifera aqueous extract **(C)** showed multifocal areas of follicular degeneration characterized with oocyte lysis in graafian follicle, degeneration of granulosa cells and theca cells, and disorganization of corona radiate and cumulus oophorus cells. Animals submitted to heat stress and receiving 100 mg/kg b.w. of *Moringa Oleifera* aqueous extract **(B)** showed a marked degeneration of graafian ovarian follicle having eosinophil amorphous material (oocyte lysis), severe degeneration of corona radiata cells, granulosa cells, and cumulus oophorus. The tissues from the animals exposed only to heat stress **(D)** showed focal areas of proliferation of fibroblast in the interstitial tissues, in addition to severe follicular degeneration characterized by oocyte lysis and accompanying enhanced general follicular cell degeneration.

## Discussion

In this study, feed consumption, body weight, and body weight gain in both pregnant and lactating female rabbits have shown to be altered with the exposition to 35–36°C. This observation is in accordance with findings of Marai et al. (35) in female rabbits submitted to heat stress under subtropical conditions of Egypt. It has been observed that in hot environmental temperatures, the appetite center of the hypothalamus is negatively affected and, therefore, decreases feed intake especially in pregnant animals prone to environmental stressors (39). The reduction in feed consumption in this study is expected since the homeostatic thermoregulatory mechanism tends to decrease endogenous heat production which might be produced by the body metabolism if feed intake is high (40) in an already heat-stressed animal. Lactating animals solely submitted to heat stress showed negative body weight gain from the 2nd week. Rhoads et al. (41) reported a decrease in feed intake by around 40% in lactating sows exposed to temperatures above 35°C where negative energy balance was observed and consequently body weight substantially decreased (42). However, in this study, administration of *Moringa oleifera* aqueous seed extract increased these parameters. The increase in feed consumption observed in this study could be explained by the presence of flavonoids found in the aqueous extract of *Moringa oleifera* seeds (16, 43). In fact, flavonoids have been implicated in protection of tissue from the destructive effects of external agents and, therefore, the structures of the central nervous system that regulate appetite (44). Stimulation of this region induces an increase in food intake, while bilateral damage induces rather a complete cessation of food intake (32).

The results of this study indicate a decrease in voluntary mating in female rabbits, an increase in number of services to achieve pregnancy, and the absence of plucking fur in female rabbits exposed to heat stress. These observations are similar to those of Marai et al. (35) in Egypt that female rabbits decreased receptivity and percentage of voluntary mating frequently in heat stress situations under subtropical conditions. Plucking fur in female rabbits means they are actively searching for materials, including their own fur to build a kid's nest, which if not done kids shall be exposed to several harmful climatic conditions that can culminate into death (35, 45). In animals, female receptivity is influenced by physiological and environmental factors such as heat stress (46). When rejecting male mating advances, female fertilization may be, at least in part, controlled by female acceptance of copulation (47).

The reduction in receptivity, voluntary mating, and plucking fur is considered as factors indicating the drop in fertility in female rabbits exposed to hot temperatures (48) and may be the cause of the low litter size reported in females submitted to heat stress in this study. Litter size has been previously linked to decreased animal fertility and conception rate with accompanying significant reduction in total young born and in an increase in percentage of young born dead in animals affected by environmental stress (45). In addition, heat stress decreases length and intensity of estrus while increasing anestrous incidence and silent heat in females (49). The improvement on litter size due to administration of *Moringa oleifera* aqueous seed extract in this study may be attributed to vitamin C and polyphenols found in Moringa (32). In this regard, these compounds have been previously reported to improve oocyte development and fertilization rate (50) by increasing distribution of compact follicular oocytes in female rabbits (51).

There was a significant decrease in litter weight from birth to weaning, individual kid body weight at weaning, and daily kid weight gain in animals exposed to heat stress. The decrease in litter weight may be associated with the reduction in food consumption in lactating female rabbits which impacts their milk production and lack or reduced nourishment of the kids. The decrease in the litter weight directly impacts other related parameters such kid body weight at weaning and daily kid weight gain. These results are similar to those observed in litter weight at 21 days by Marai et al. (6) and at 35 days by Marai et al. (35). The amelioration of these parameters, following the administration of *Moringa oleifera* aqueous seed extract in this study, has also been demonstrated by El-Desoky et al. (52) in female rabbits receiving, respectively, 10, 25, and 50 mg/kg body weight of nonencapsulated *Moringa oleifera* leaves ethanolic extract during summer seasons. Moreover, various phenol compounds and vitamin C found in *Moringa oleifera* have the ability to prevent the digestive system cell membranes from oxidative stress by scavenging O_2_ radicals and, therefore, restoring feed utilization (53). This action mechanism by these compounds may partly explain the rise in pre-weaning weight gain, as well as the higher weight at weaning and the decrease in pre-weaning deaths (54). Indeed, in this study, kids' weight is strongly correlated with milk production, milk intake, and milk efficiency. The results demonstrate that the low energy intake from food consumption is not enough to cover for both animal body metabolism and daily requirements for milk production (39). It can be argued that, on the one hand, the low daily milk production reported in this study is probably due to the decrease in feed consumption as a result of body mechanism to decrease heat production and, on the other hand, heat stress reduced milk production by decreasing mammary cell proliferation (55). The drop in litter weight week after week is the result of the observed low milk production as kids become more and more exigent in milk quantity for their metabolic needs, and this situation leads to the daily milk efficiency of converting milk into body weight gain. The improvement of these parameters could be associated with the antioxidant activity of bioactive compounds present in *Moringa oleifera* that act against lipid oxidation in the cell membrane (14, 56). This is also important in pre-weaning kids which have been reported to be highly sensitive to oxidative stress than adults and for the development of the immune system in young animals (57).

The elevated daily milk intake in litter submitted only to heat stress compared to those receiving *Moringa oleifera* and the control group is mainly due to the high kids' mortality rate reported in this group. It is noted that at weaning, there were only 28.6% kids alive in the group submitted to heat stress alone compared to 83.1% of the control group. Therefore, the little quantity of milk produced by these females was enough to meet kids' requirements in contrast to animals of the control group that produced a large quantity of milk but insufficient to meet kids' nutritional needs.

All these reported parameters are under the influence of reproductive hormones as their main function is to regulate reproductive system and therefore ensure the success of such reproductive event (14). An increase in ambient temperature of more than 2°C is responsible for low or desynchronized endocrine activities in females, mainly the pineal-hypothalamo-hypophyseal-gonadal axis, followed by the impairment of the respective reproductive hormone functions (58). A deterioration of reproductive hormones (LH, FSH, estradiol, and progesterone) and stress hormone (cortisol) following exposition to heat stress was observed in this study. Heat stress is responsible for the impairment of the follicular estradiol synthesis activity and the reduction of LH activity by decreasing its receptor level (59). The decrease in the fertilization rate due to the inhibition of estrus signs, gonadotrophin decrease, and ovulation disruption has been associated with low estradiol secretion (60). Moreover, the decrease in FSH serum content in heat-stressed female rabbits is mostly a consequence of decrease in the inhibition of negative feedback from smaller follicles which finally impair the animal reproductive efficiency (61). The low progesterone production observed in heat-stressed female rabbits impairs the embryo development by disrupting endometrial function (60, 61), while acyclicity and infertility as well as mammary gland development impairment in pregnant females have been associated with the increased prolactin in heat-stressed females (49). Cortisol is a stress hormone released by the adrenal gland and is important in enabling the body to deal with stressful situations by enhancing blood sugar production, converting fats, proteins, and carbohydrates into usable energy (62). However, an increase in levels of these hormones in this study has been observed following administration of *Moringa oleifera* aqueous seed extract. Several compounds found in this plant have been reported to stimulate reproductive hormones production. For instance, phytosterols have a chemical structure similar to that of cholesterol that can be used as precursors of steroid hormones (testosterone, estradiol, and progesterone), while isoflavones are one of the flavonoid compounds, also have estrogenic activity, and are able to bind with estrogen receptors such as ER-α and ER-β (63). Phenolic compounds and alkaloids have been reported to protect embryonic tissue against reactive oxidative stress impairment by enhancing the ovarian hormones serum concentration (64). Phytosterols, polyphenols, saponins, and flavonoids present in *Moringa oleifera* seeds have been previously associated with the increase in female reproductive hormones (65, 66).

Ovary histology is usually used to assess its morphological changes leading to cells and reproductive hormone impairment (67). Analysis of the micrographs showed damages of ovary of female rabbits solely exposed to heat stress. These histopathologies were characterized by focal areas of proliferation of fibroblast in the interstitial tissues evidenced by the presence of large opened spaces. However, the administration of *Moringa oleifera* aqueous seed extract showed minimal alterations with atretic follicles.

## Conclusion

The aqueous extract of the seeds of *Moringa oleifera* minimizes the adverse effects of heat stress inducing reproductive impairments in female rabbits mainly due to a large variability of its bioactive compounds thus providing this plant with a high antioxidant activity. From the foregoing, *Moringa oleifera* aqueous seed extract could be used as a mitigation measure for managing heat stress and associated complications in rabbit farming with sole aim of improving on reproductive performance and production.

## Data availability statement

The raw data supporting the conclusions of this article will be made available by the authors, without undue reservation.

## Ethics statement

The animal study was reviewed and approved by the Ethical Committee of the Faculty of Veterinary Medicine, University of Nairobi (REF: FVM BAUEC/2019/244).

## Author contributions

AN, JK, and JM supervised and designed the project, cross checked the draft of the manuscript, and finally approved for submission. VM designed the project, conducted the experiment, collected, analyzed data, and wrote the first draft of the manuscript. RA and CB assisted in the conduction of the experiment and collected data. VM and RA conducted laboratory analysis of experiment. VM, AN, RA, VM, and CB rechecked the draft of the manuscript. All authors contributed to the article and approved the submitted version.

## Funding

This work was funded by the Université Evangélique en Afrique (UEA) through the University project on improvement of research and teaching quality funded by Pain pour le Monde (grant number: A- COD- 2018- 0383).

## Conflict of interest

The authors declare that the research was conducted in the absence of any commercial or financial relationships that could be construed as a potential conflict of interest.

## Publisher's note

All claims expressed in this article are solely those of the authors and do not necessarily represent those of their affiliated organizations, or those of the publisher, the editors and the reviewers. Any product that may be evaluated in this article, or claim that may be made by its manufacturer, is not guaranteed or endorsed by the publisher.
